# Clinical features, management and visual outcome of polypoidal choroidal vasculopathy in Indian patients

**DOI:** 10.4103/0301-4738.67052

**Published:** 2010

**Authors:** Giridhar Anantharaman, Gudapati Ramkumar, Mahesh Gopalakrishnan, Alpesh Rajput

**Affiliations:** Department of Vitreoretinal Services, Giridhar Eye Institute, Cochin, India

**Keywords:** Greatest linear diameter, indocyanine green angiography, photodynamic therapy, polypoidal choroidal vasculopathy, serosanguinous maculopathy, submacular hemorrhage, thermal laser

## Abstract

**Aims:**

To present the clinical, indocyanine green angiography (ICGA) features and results of treatment for polypoidal choroidal vasculopathy (PCV) in Indian patients by a retrospective chart review.

**Materials and Methods:**

Forty five patients with PCV underwent complete ocular examination, fluorescein angiography (FFA) and ICGA. Treatment was advised for patients with macular involvement and progressive loss of visual acuity. Demographic data, clinical features and results of treatment were analyzed.

**Results:**

Mean age at presentation was 61.06 years. Mean follow up was 18 months. The disease was more prevalent in males. Forty three patients had unilateral disease. The most common location of polyps in ICGA was subfoveal (42.5%). Exudative form was seen in 34 of the 47 eyes and the remaining 13 eyes had a hemorrhagic presentation. Thirty four eyes underwent treatment which included thermal laser (*n* = 11), photodynamic therapy (PDT) (*n* = 11) and transpupillary thermo therapy (TTT) (*n* = 12). Statistical analysis was done using the Chi-square test. Subgroup analysis of visual outcome following various modalities of treatment showed that the results of PDT (*P* < 0.001) and thermal laser (*P* < 0.001) were statistically significant.

**Conclusions:**

PCV is an important differential diagnosis in patients presenting with serosanginous maculopathy and submacular hemorrhage. The disease was more prevalent in males and was unilateral in the Indian population. Timely intervention in cases with symptomatic polyps could achieve stabilization of visual acuity. Thermal laser and PDT were safe and effective.

Polypoidal choroidal vasculopathy (PCV), first described by Yanuzzi *et al*.[1] in 1990, is a distinct clinical entity characterized by persistent, recurrent serous leakage and hemorrhage in the macula, and is seen in the elderly population. The disorder was poorly understood earlier and initially designated as recurrent retinal pigment epithelial detachments (PED) and posterior uveal bleeding syndrome.[2–5] The primary abnormality as described by Yanuzzi *et al*.[1,2,6] involves the choroidal circulation, and the characteristic lesion is an inner choroidal vascular network of vessels ending in an aneurysmal bulge or outward projection, visible clinically as a reddish orange spheroidal, polyp like structure.[7] The exact pathogenesis of these vascular abnormalities is still not known. Indocyanine green angiography (ICGA) is essential for the diagnosis as it permits visualization of the choroidal vasculature with enhanced specificity and sensitivity.[6]

Several reports have suggested that the incidence of PCV is markedly high in black people, relatively high in Asian population and low in white people in contrast to age-related macular degeneration.[3–5,7,8] In blacks, PCV affects women more frequently and is usually associated with peripapillary involvement.[2–4,8,9] Several reports from the Asian population have shown a male preponderance, unilateral presentation with macular involvement.[5,6,8,9] Many different treatment modalities have been used to preserve vision in patients with symptomatic PCV. Direct laser photocoagulation,[10,11] feeder vessel treatment,[12] transpupillary thermo therapy (TTT)[13] and photodynamic therapy (PDT)[14–21] are some of the modalities which have been described.

The aim of this study is to describe the demographic, clinical features and results of treatment in 45 patients with PCV from the Indian subcontinent.

## Materials and Methods

For this interventional case study, we retrospectively reviewed 45 consecutive patients with PCV who visited the retina clinic of our institute for the first time between January 2005 and March 2008. All the patients underwent a complete ophthalmic evaluation, indirect ophthalmoscopy, slit lamp biomicroscopy, color fundus photography, fundus fluroscein angiography (FFA) and ICGA. The diagnosis of PCV was based on ICGA performed using the Carl Zeiss Visupac system. The features that were studied included the type and location of the polyps and the change in lesion characteristics following treatment. Polyps were localized to four areas according to the ICGA findings: peripapillary (within one disk diameter of the optic disc), subfoveal, juxta foveal (within 200 μm from the center of fovea) and extrafoveal (beyond 200 μm from the center of the fovea). Based on clinical presentation, we classified the eyes into two types: (1) exudative form characterized predominantly by the presence of one or all of the following (i) intraretinal lipid deposits, (ii) serous macular detachment, (iii) serous PED and (2) hemorrhagic form characterized predominantly by the presence of one or all of the following (i) subretinal hemorrhage (ii) hemorrhagic PED involving the macula.

Treatment options included thermal laser, TTT and PDT using verteporfin. Polypoidal lesions seen on ICGA, which were outside the foveal avascular zone, were treated with thermal laser using the 532-nm green laser, 100–200 μm spot size at 0.2–0.3 second interval. The endpoint of treatment was whitening of the area of the polyp. Thermal laser was combined with intravitreal antivascular endothelial growth factor (VEGF) injection into eyes with significant serosanginous maculopathy. Subfoveal polyps and peripapillary polyps within papillomacular bundle were treated with ICGA guided PDT. This was combined with intravitreal anti-VEGF, if necessary, to reduce the exudation and hemorrhage. PDT with verteporfin (Visudyne, Novartis. Basal, Switzerland) was administered as per the treatment of age-related macular degeneration using PDT (TAP) study protocol.[22] For patients undergoing PDT, greatest linear diameter (GLD) was determined using ICGA angiography to cover the entire area of all leaking polyps and the adjoining vascular network. The diameter of laser spot size was determined by adding 1000 μm to the GLD. ICGA angiography was repeated 3 months after treatment. TTT was performed for subfoveal or juxta foveal polyps in patients who could not afford PDT. The laser spot size for TTT was calculated so as to cover the area of the polyps and the abnormal vascular network based on ICGA, and the power was calculated based on a test burn. All the treated patients were examined at 3 months when best-corrected visual acuity, ophthalmoscopic examination and ICGA were performed. Subsequent examination was performed every 3 months during the period of follow up. Associated relevant systemic diseases (e.g. hypertension, diabetes) were identified by either the patient’s history or his/her use of systemic medications.

Statistical analysis was done using Chi-square test. Visual acuity was recorded with Snellen’s chart and converted to LogMAR for statistical analysis. Data were expressed in mean and discrete data were expressed as numbers (*N*) and percentages (%). Chi-square test was applied for discrete data. For all statistical tests, the significance level was taken as *P* < 0.05.

## Results

Forty seven eyes of 45 patients were eligible for the study. Mean age of the patients was 61.06 years (41–80 years) and mean follow-up was 18 months (3–39 months). The disease was more prevalent in males (M:F = 1.4:1). Two patients had bilateral disease while the remaining 43 patients had unilateral involvement.

**Figure 1:**
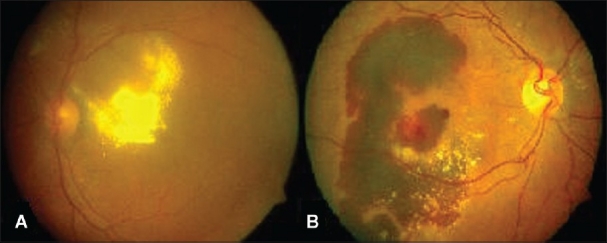
(A) Exudative pattern with extensive intraretinal lipid deposits in the macula. (B) Hemorrhagic pattern with large subretinal hemorrhage extending to the centre of the macula

The most common clinical presentation was a gradual loss of vision in 32/45 patients (70%) with a mean duration of 3.69 months. (1–12 months). The remaining 13 persons presented with sudden loss of vision with a mean duration of 5.7 days (1–14 days). Mean size of lesion on presentation was 4.56 disk diameters (1–10 disk diameter). Exudative pattern [Fig. 1A] was seen in 34 eyes while the remaining 13 eyes had a predominantly hemorrhagic form [Fig. 1B]. Serous macular detachment and serous PED were the most common clinical findings in the macula. Six eyes with hemorrhagic pattern had associated intraretinal lipid deposits.

In patients with unilateral disease, disciform scarring was seen in six fellow eyes. Two eyes had retinal pigment epithelial atrophy in the macula and one eye had myopic macular degeneration. Based on the location of the polyps in ICGA, they were classified as extrafoveal (8 eyes), subfoveal (19 eyes), peripapillary (14 eyes) and juxta foveal (8 eyes). Three patients had polyps in multiple locations in the macula and most patients (39/47) had polyps in cluster.

**Figure 2:**
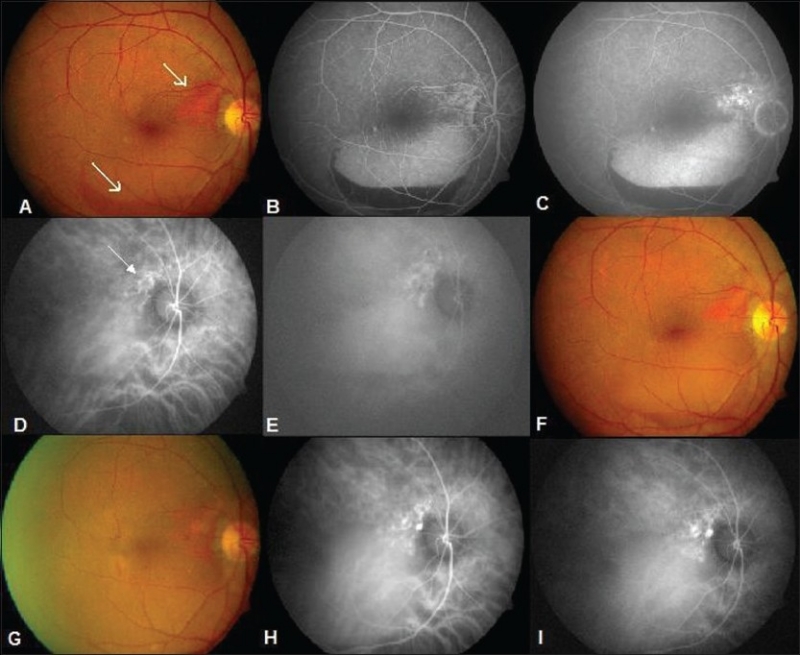
(A 62-year-old male with 20/30 vision (A) serosanguinous maculopathy (arrow) with subretinal hemorrhage (arrow). Note reddish orange elevation in the peripapillary region (arrow). (B and C) Early and late FFA shows a serous PED. Note blocked fluorescence in the inferior edge of the PED suggestive of a hemorrhage. (D and E) ICGA pictures showing saccular dilatations in the peripapillary region (arrow). (F) 12 months later clinical picture shows no change. (G) 36 months after the initial examination, fundus photograph shows less hemorrhage and fluid. (H and I) Repeat ICG however shows no change

Thirty four of 47 eyes which underwent treatment were eligible for analysis of treatment outcomes. Of the remaining 13 eyes, 4 had a visual acuity of 20/30 or better and therefore not subjected to any form of treatment. The natural course of these four patients was favorable and one of these patients has a follow up of 36 months with stable visual acuity and no intervention[Fig. 2]. In the two patients with bilateral disease, only one eye was treated as the fellow eye had advanced disease with very poor visual outcome. The remaining seven patients did not report for treatment. Pneumatic displacement was performed initially at the time of clinical presentation for submacular hemorrhage in six eyes.

**Figure 3:**
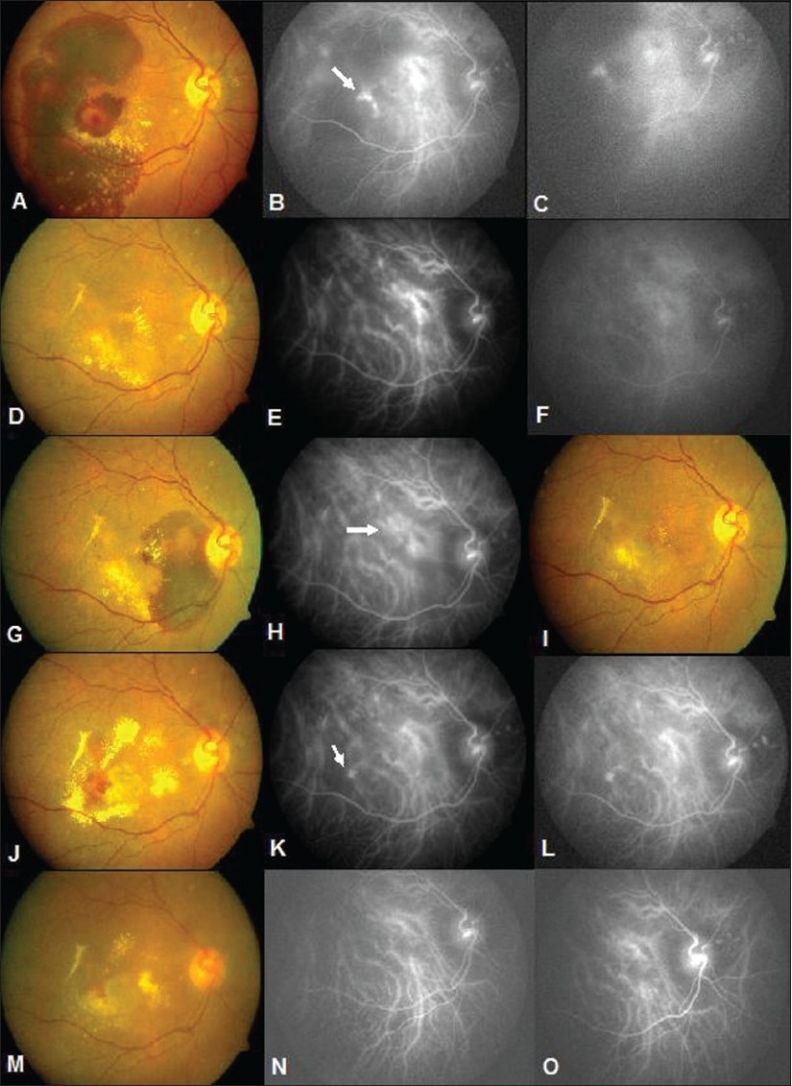
A 64-year-old female. (A) Note submacular hemorrhage. Visual acuity 20/120. (B and C) ICGA shows saccular dilatations in cluster. (D) 3 months following PDT note resolution. (E and F) ICGA shows regression of polyps. (G) Seven months later presents with recurrence. (H) ICGA demonstrates recurrence of polyps. (I) 3 months following PDT note the absorption of blood. (J) 17 months later presented with intraretinal lipid deposits in the macula and reduced vision. (K and L) ICG shows extrafoveal polyps. (M) Following thermal laser intraretinal lipids decreased. (N and O) ICGA shows regression of polyps (20/80)

Eleven patients were treated with PDT. The demographic features and results of treatment are described in Table 1. Mean follow up was 15.9 months (3–39 months). Three eyes received intravitreal bevacizumab and one eye received intravitreal triamcinolone acetonide along with PDT. The purpose of combining intravitreal pharmacotherapy with PDT was to reduce the significant exudation. All the treated patients showed significant improvement in the form of resolution of exudates and hemorrhage at 3 months after regression of polyps in ICGA. Visual acuity improved in nine eyes, worsened in one eye and remained the same in one eye. A representative case is illustrated in Fig. 3.

**Table 1:** Photodynamic therapy for polypoidal choroidal vasculopathy

Age/sex	Duration of symptoms (days)	Type of clinical presentation	Visual acuity at first visit (logMAR)	Visual acuity at final visit (logMAR)	Laser spot size	Retreatment	Duration of follow up (months)
41/F	7	Hem	1.8	1.3	2150		15
48/M	30	Hem	0.5	0.0	2110		12
57/M	7	Exudates	1.6	1.3	2440		9
60/F	30	Exudates	1	0.8	3960		28
60/F	120	Exudates	0.5	0.3	2250		13
76/M	30	Hem + exudates	0.8	0.5	1700	PDT	9
64/F	120	Exudates + hem	0.5	0.8	3450	PDT, thermal laser	36
60/F	180	Exudates	1.8	0.8	2200		12
58/F	4	Hem	1.8	0.5	1660	PDT	34
52/F	270	Exudates	0.5	0.2	3400		3
61/F	90	Exudates	0.3	0.0	2830		3

F: Female, M: Male, hem: hemorrhage

Three patients had recurrence of polypoidal lesions at the edge of the previously treated lesion and presented with recurrence of symptoms in the form of decreased visual acuity and clinically reappearance of hemorrhage and exudation. Recurrences were noted at 7 months following the initial treatment in one eye, at 33 months in the second patient and at 25 months in the third patient. One of these three patients had a second recurrence 17 months following the first recurrence Fig. 3. Mean pretreatment visual acuity of all the treated patients improved from 0.95 logMAR units to a post-treatment visual acuity of 0.59 logMAR units (*P* < 0.001) which was statistically significant.

**Figure 4:**
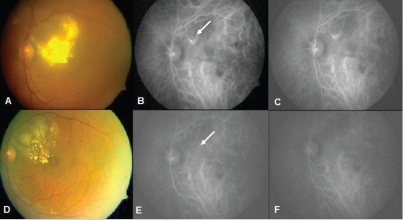
A 68-year-old male presented after receiving three injections of intravitreal bevacizumab elsewhere with no response and further decrease in vision. (A) Initial visit shows intraretinal lipid deposits involving the fovea. Visual acuity 20/80. (B and C) Early and late ICGA shows saccular dilatation extrafoveal in location (arrow). (D) Seven months following laser, clinical photograph shows significant decrease in intraretinal lipid deposits and visual acuity improved to 20/30. (E and F) Post-treatment ICGA shows regression of the polyps

Table 2 gives the details of the 11 patients treated with thermal laser. Mean follow up was 10.36 months (average 3–36 months). Visual acuity improved in 10 eyes and worsened in 1 eye. There was a significant improvement in the clinical picture following treatment and post-treatment. ICGA showed closure of polyps in all the eyes. A representative case is illustrated in Fig. 4. Mean pretreatment visual acuity improved from 1.18 logMAR units to a post-treatment visual acuity of 0.28 logMAR units, a difference that was significant (*P* < 0.001). The treatment was safe with no adverse events. Recurrence was noticed in one eye, 6 months following treatment, and this was retreated with thermal laser. Intravitreal bevacizumab was combined with thermal laser in two eyes with significant exudation.

**Table 2:** Thermal laser for polypoidal choroidal vasculopathy

Age/sex	Duration of symptoms (days)	Type of clinical presentation	Visual acuity at first visit (logMAR)	Visual acuity at final visit (logMAR)	Combined with intravitreal avastin	Duration of follow up (months)
57/F	7	Exudates	1.3	0.6		3
68/M	240	Exudates	0.5	0.0		6
62/M	7	Hem	0.5	0.2		36
80/M	30	Exudates	0.3	0.5	Yes	12
68/M	150	Exudates	0.5	0.0	Yes	8
60/F	4	Hem	1.6	0.2		14
62/M	7	Exudates	1.3	0.2		36
58/M	2	Exudates	0.8	0.3		3
71/M	14	Hem	1.8	0.5		22
63/F	180	Exudates	0.5	0.2		7
66/F	30	Exudates	0.5	0.3		3

Of the 12 patients treated with TTT, visual acuity improved in four eyes, remained the same in five eyes and worsened in the remaining three eyes. Mean follow up was 12.5 months. The adverse effect of treatment included massive subretinal hemorrhage (one eye) and retinal pigment epithelial atrophy (four eyes). Persistence of clinical signs in the form of exudation and hemorrhage was seen in three eyes. Mean visual acuity improved from 1.07 logMAR units to a post-treatment visual acuity of 0.79 logMAR units (*P* = 0.003). Five of the 12 eyes required retreatment.

## Discussion

In the present study, the mean age of patients was 61.06 years which is comparable to other studies of Asian patients with PCV.[23–27] The male female ratio of 1.4:1 observed in this study further corroborates the fact that PCV has a male predilection in Asian population[5,23,24] unlike the Caucasians wherein there is a female preponderance.[2] The bilateral involvement of 4.5% in this series is less than that reported in other Asian ethnic groups (24% in Koreans,[14,22] 10% in Japanese cohorts).[5] The high incidence of macular polyps (74.5%) in this series is again consistent with the observations made in other Asian ethnic groups.[5,10,11,14]

Retinal manifestation of PCV resembles those of wet age-related macular degeneration. ICGA is essential for the diagnosis and in its absence many of these patients would be managed as wet AMD.[6] Characteristically, in this series PCV was not associated with wet AMD, suggesting that the pathogenesis of PCV differs from that of AMD. However, examination of fellow eyes of few patients showed disciform scar (six eyes) and RPE atrophy (two eyes), changes that are seen in age-related macular degeneration. It is also possible that these changes are secondary to long standing serous macular detachment or submacular hemorrhage secondary to PCV resulting in RPE atrophy and disciform scar, respectively. Similar observations have been made by Uyama *et al*.[28] in their study on the ‘Natural history of PCV’ in 14 eyes over a period of 2 years.

The criteria for selecting a treatment modality were based on the location of the polyps. Extrafoveal polyps were treated with thermal laser. Peripapillary and subfoveal polyps were treated with either PDT or TTT depending on the affordability of the patient. Numerous reports have been published concerning the results of PDT for PCV.[13–21] In all of these reports, with a minimum follow up of 1 year, preservation or improvement of visual acuity was achieved in 80–100% of patients. In our series, stabilization or improvement was noticed in 90% of patients with a mean follow up of 15.9 months. Seven of 11 patients in our series had a follow up of at least 1 year and 3 of these patients who had a follow up of more than 2 years had recurrence of polyps. In a large series of 47 eyes with a follow up of 24 months, Azara *et al*.[19] reported a recurrence of polypoidal lesion in 30 of 47 eyes followed for 24 months or more after the first PDT. However, the percentage of eyes that remained visually stable was 80% in the recurrence group. The incidence of polypoidal lesion recurrence increases with longer duration of follow up.[18] In our series, only 3 of the 11 eyes had a follow up of more than 2 years and therefore we need a longer follow up to comment on the long-term effects of PDT in PCV. We have used ICGA guided PDT for deciding the GLD of the lesion in all our cases. This was first reported by Chan *et al*.[13] The GLD of the lesion on ICGA is usually smaller than that on FFA, so ICGA guided PDT for PCV may reduce the risk of damaging the normal choroidal vasculature included in the laser spot, thereby reducing the complications.[18] We did not encounter any adverse events following treatment, although there are reports of hemorrhagic complications following PDT.[20,21]

There are few published reports on the role of thermal laser for PDT and the results are variable.[10,11] Lee *et al*.[10] showed stabilization or improvement of vision in 18 out of 23 eyes with extrafoveal polyps at 12 months with recurrence in 2 eyes. In our series, the visual results were statistically very significant with 9 of 11 eyes showing visual improvement and there was recurrence only in one eye. There was no development of choroidal neovascularization, as has been reported, during the period of follow up in our series.[10]

In a recent report, Mitamura *et al*.[13] compared the results of PDT to those of TTT in patients with PCV and they found that the visual results of the PDT group at 12 months (*P* = 0.0006) was significantly better than those of the TTT group. Two patients in the TTT group had vitreous hemorrhage following treatment, whereas there was no adverse event in the PDT treated eyes. In our study, the visual results of the PDT group were far better than those of the eyes treated with TTT. Whereas all the patients treated with PDT showed significant clinical improvement at 3 months, only 3 out of the 12 eyes treated by TTT showed significant clinical improvement during the period of follow up. Moreover, TTT was associated with massive subretinal hemorrhage in one eye. We therefore feel that PDT is a safer and more effective treatment although randomized trial will be necessary to compare the long-term safety and efficacy of these two treatments in PCV. The purpose of this article is to highlight the various clinical presentations and different management options in PCV. However, in view of its retrospective design, this study has its limitations. This includes small number of cases, different baseline characteristics, variable follow up, and also, the treatment is not designed uniformly and cannot be compared.

To our knowledge, this is the largest series of patients with PCV reported from India. The demographic profile, laterality and clinical features are similar to other Asian ethnic groups. Treatment of PCV is indicated in patients with symptomatic polyps with macular involvement. Thermal laser and PDT were found to be safe and effective treatments. Timely intervention does result in stabilization of visual acuity in a large percentage of patients.

## References

[1] Yannuzzi LA, Sorenson J, Spaide RF, Lipson B (1990). Idiopathic polypoidal choroidal vasculopathy (IPCV). Retina.

[2] Yannuzzi LA, Ciardella A, Spaide RF, Rabb M, Freund KB, Orlock DA (1997). The expanding clinical spectrum of idiopathic polypoidal choroidal vasculopathy. Arch Ophthalmol.

[3] Stern RM, Zakov ZN, Zegarra H, Gutman FA (1985). Multiple recurrent serosanguineous retinal pigment epithelial detachments in black women. Am J Ophthalmol.

[4] Kleiner RC, Brucker AJ, Johnston RL (1990). The posterior uveal bleeding syndrome. Retina.

[5] Sho K, Takahashi K, Yamada H, Wada M, Nagai Y, Otsuji T (2003). Polypoidal choroidal vasculopathy: Incidence, demographic features, and clinical characteristics. Arch Ophthalmol.

[6] Spaide RF, Yannuzzi LA, Slakter JS, Sorenson J, Orlach DA (1995). Indocyanine green video angiography of idiopathic polypoidal choroidal vasculopathy. Retina.

[7] Moorthy RS, Lyon AT, Rabb MF, Spaide RF, Yannuzzi LA, Jampol LM (1998). Idiopathic polypoidal choroidal vasculopathy of the macula. Ophthalmology.

[8] Ciardella AP, Donsoff IM, Huang SJ, Costa DL, Yannuzzi LA (2004). Polypoidal choroidal vasculopathy. Surv Ophthalmol.

[9] Gomi F, Tano Y (2008). Polypoidal choroidal vasculopathy and treatments. Curr Opin Ophthalmol.

[10] Lee MW, Yeo I, Wong D, Ang CL (2009). Argon laser photocoagulation for the treatment of polypoidal choroidal vasculopathy. Eye (Lond).

[11] Ladas ID, Karagiannis DA, Georgalas I, Rouvas AA, Moschos MM, Apostolopoulos M (2004). Polypoidal choroidal vasculopathy associated with Doyne’s familial choroiditis: Treatment with thermal laser. Eur J Ophthalmol.

[12] Lai TY, Chan WM, Lam DS (2004). Laser photocoagulation of indocyanine green angiographically identified feeder vessels to idiopathic polypoidal choroidal vasculopathy. Am J Ophthalmol.

[13] Mitamura Y, Kubota-Taniai M, Okada K, Kitahashi M, Baba T, Mizunoya S (2009). Comparison of photodynamic therapy to transpupillary thermotherapy for polypoidal choroidal vasculopathy. Eye (Lond).

[14] Chan WM, Lam DS, Lai TY, Lui DT, Li KK, Yao Y (2004). Photodynamic therapy with verteporfin for symptomatic polypoidal chroidal vasculopathy: One year results of a prospective case series. Ophthalmology.

[15] Spaide RF, Donsoff L, Lam DL, Yannuzzi LA, Jampol LM, Slakter J (2002). Treatment of polypoidal choroidal vasculopathy with photodynamic therapy. Retina.

[16] Hussain N, Hussain A, Natarajan S (2005). Role of photodynamic therapy in polypoidal choroidal vasculopathy. Indian J Ophthalmol.

[17] Gomi F, Ohji M, Sayanagi K, Sawa M, Sakaguchi H, Oshima Y (2008). One year outcomes of photodynamic therapy in age-related macular degeneration and polypoidal choroidal vasculopathy in Japanese patients. Ophthalmology.

[18] Silva RM, Figueira J, Cachulo ML, Duarte L, Faria de Abreu JR, Cunha-Vaz JG (2005). Polypoidal choroidal vasculopathy and photodynamic therapy with verteporfin. Graefes Arch Clin Exp Ophthalmol.

[19] Akaza E, Mori R, Yuzawa M (2008). Long term results of photodynamic therapy of polypoidal choroidal vasculopathy. Retina.

[20] Hirami J, Tsujikawa A, Otani A, Yodoi Y, Aikawa H, Mandai M (2007). Hemorrhagic complications after photodynamic therapy for polypoidal choroidal vasculopathy. Retina.

[21] Rishi P, Kadekar A, Rishi E (2009). Breakthrough vitreous hemorrhage after ICGA guided PDT for PCV. Indian J Ophthalmol.

[22] (1999). Photodynamic therapy of subfoveal choroidal neovascularization in age-related macular degeneration with verteporfin: One year results of 2 randomized clinical trials: TAP report. Treatment of age related macular degeneration with photodynamic therapy (TAP) Study Group. Arch Ophthalmol.

[23] Byeon SH, Lee SC, Oh HS, Kim SS, Koh HJ, Kwon OW (2008). Incidence and clinical patterns of polypoidal choroidal vasculopathy in Korean patients. Jpn J Ophthalmol.

[24] Wen F, Liu Y, Huans S (2004). Polypoidal choroidal vasculopathy in elderly Chinese patients. Graefes Arch Clin Exp Ophthalmol.

[25] Kwok AK, Lai TY, Chan CW, Neoh EL, Lam DS (2002). Polypoidal choroidal vasculopathy in Chinese patients. Br J Ophthalmol.

[26] Uyama M, Matsubara T, Fukushima I, Matsunaga H, Iwashita K, Nagai Y (1999). Idiopathic polypoidal choroidal vasculopathy in Japanese patients. Arch Ophthalmol.

[27] Cackett P, Wong D, Yeo I (2009). A classification system for polypoidal choroidal vasculopathy. Retina.

[28] Uyama M, Wada M, Nagai Y, Matsubara T, Matsunaga H, Fukushima I (2002). Polypoidal choroidal vasculopathy: Natural history. Am J Ophthalmol.

